# Mortality Related to Pneumonia and Diabetes Mellitus: A Retrospective Study

**DOI:** 10.7759/cureus.89169

**Published:** 2025-07-31

**Authors:** Krithika V, Dency D Mavani, Diya V Patel, Gayathri Dantu, Sai Praneeth Chaparala, Hyoyoung Song

**Affiliations:** 1 Internal Medicine, Stanley Medical College, Chennai, IND; 2 Internal Medicine, Smt. Nathiba Hargovandas Lakhmichand Municipal Medical College, Ahmedabad, IND; 3 Internal Medicine, Government Medical College, Mahabubnagar, Mahabubnagar, IND; 4 Internal Medicine, Gayatri Vidya Parishad Institute of Health Care and Medical Technology, Visakhapatnam, IND; 5 Family Medicine, Dr. Na Family Medicine Clinic, Seoul, KOR

**Keywords:** age-adjusted mortality rate, cdc, diabetes mellitus, mcd, pneumonia, retrospective study

## Abstract

Introduction: Pneumonia is a major cause of mortality, and its association with diabetes mellitus (DM) remains underexplored. Understanding this relationship is essential to identifying high-risk populations and developing targeted public health interventions.

Aim: To analyze mortality trends and demographic disparities in pneumonia with DM as a contributing cause using the Centers for Disease Control (CDC) Multiple Causes of Death (MCD) database from 1999 to 2020.

Methods: A retrospective observational study was conducted using the CDC MCD database to assess mortality trends in individuals aged 25 years and older in the United States from 1999 to 2020. The study included deaths where pneumonia (International Classification of Diseases-10 (ICD-10): J18) was listed as the underlying cause and DM (ICD-10: E10-E14) as a contributing cause. Data were analyzed by age, gender, race, geographic region, and place of death. Age-adjusted mortality rates (AAMR) and annual percentage change (APC) were calculated.

Results: A total of 59,291 deaths were recorded. The AAMR for pneumonia with DM as a contributing cause initially showed a rising trend from 1999 to 2002 with an APC of 3.90% (p < 0.05). However, from 2002 to 2018, the AAMR began declining significantly with an APC of -4.56% (p < 0.05). Deaths were more common in females (51.1%) and White individuals (82.0%). Most deaths occurred in metropolitan areas (79.5%) and medical facilities (70.9%).

Conclusion: These findings highlight the need for integrated DM management and pneumonia prevention strategies. Targeted vaccination programs and early respiratory infection management could help reduce mortality in high-risk groups.

## Introduction

Pneumonia, commonly caused by respiratory viruses, gram-negative or gram-positive bacteria, and, internationally, mycobacteria, is the leading cause of acute lung injury (ALI) and acute respiratory distress syndrome (ARDS). Though the chances of fungal or parasitic infections rise in immunocompromised people, pneumonia is less often caused by these pathogens [1]. Pneumonia involves lung inflammation affecting air sacs and tissue, triggering an immune response and causing fever, cough, breathlessness, and chest pain. Complications can include organ failure and lasting impacts on quality of life and lung function [2]. In 2022, pneumonia in the US accounted for 41,108 deaths (12.3 per 100,000), and 1.4 million ED visits were reported by the Centers for Disease Control and Prevention (CDC), with potential underestimation of the true burden [3]. Pneumonia incidence is higher in men, peaking in the very young and old. Mortality increases with age, is elevated in rural US areas and some states, and disproportionately affects Black American individuals [3,4].

Diabetes mellitus (DM) encompasses primarily type 2 DM (T2DM) (90-95% of cases), characterized by insulin resistance and deficiency, and type 1 DM (T1DM) (5-10%), marked by autoimmune destruction of pancreatic beta-cells. Gestational diabetes occurs in some pregnancies, while secondary diabetes is linked to other conditions [5]. A study by Pan et al. showed a bidirectional relationship between bacterial pneumonia and gestational diabetes, and also with T1DM [6]. Pneumonia patients with T2DM and higher admission HbA1c, prior insulin usage, or more comorbidities (Charlson Comorbidity Index, CCI) had poorer in-hospital glucose control. Higher CCI also raised readmission risk, while greater acute illness increased mortality risk [7]. In addition to promoting chronic inflammation and antibiotic resistance, DM compromises immunity (monocyte/neutrophil activity, inflammation, and complement), and hyperglycemia directly erodes defences. The likelihood and severity of infections, potentially pneumonia, are further increased by coexisting disorders [8].

The CDC is the leading public health agency focused on protecting health and safety. It monitors diseases, investigates outbreaks, conducts research, develops guidelines, educates the public, collaborates with health departments globally, and responds to health emergencies, all with a focus on prevention [9]. This study aims to assess the link between DM and pneumonia mortality, as DM may increase the risk and severity of pneumonia, leading to worse outcomes. Further research is needed to confirm this association and understand the underlying biological mechanisms for targeted interventions.

The aim of the study is to analyze mortality trends from 1999 to 2020 among patients aged 25 years and older with both pneumonia and DM, using the CDC Multiple Causes of Death (MCD) database, stratified by sex, race, level of urbanisation, and place of death.

## Materials and methods

A retrospective original research study was conducted using the CDC Wide-Ranging Online Data for Epidemiologic Research (CDC WONDER) MCD database [9]. The study utilized publicly available mortality data, which includes de-identified death certificate information for all deaths recorded in the US. Data extraction was performed on April 14, 2025, and as the dataset consists of publicly available, de-identified information, the study was classified as non-human participant research, thus exempt from institutional review board (IRB) approval.

Mortality data were extracted from the CDC WONDER MCD database for the years 1999-2020. The study included individuals aged 25 years and above. Pneumonia (J18) was selected as the underlying cause of death, while DM (E10-E14) was selected as the multiple cause of death to assess the co-occurrence of these conditions. Demographic variables such as gender (male and female) and race/ethnicity (American Indian or Alaska Native, Asian or Pacific Islander, Black or African American, White) were included to analyze disparities in mortality outcomes. Geographic variables included urbanization based on the 2013 classification, categorizing metropolitan cities into large central metro, large fringe metro, medium metro, and small metro, and non-metropolitan cities into micropolitan and non-core rural areas. Additionally, the place of death was categorized as a medical facility, home, hospice, or nursing facility. Mortality rates were standardized using age-adjusted rates per 1,000,000 population, with adjustments based on the US Standard Population from the year 2000 to allow for accurate comparisons over time.

Descriptive statistics, including absolute numbers and percentages, were used to summarize the demographic and geographic variables. Those not meeting the above variables were excluded from the study. Age-adjusted mortality rates (AAMRs) were calculated for each subgroup using the CDC WONDER MCD database. To evaluate temporal trends, Join Point Regression Analysis (Join Point Software Version 5.3.0.0, November 2024; Surveillance Research Program, National Cancer Institute, Bethesda, MD, USA) was used to determine annual percentage changes (APC) in pneumonia-related mortality with DM as a contributing cause. Trends were assessed over the 1999-2020 study period to identify statistically significant changes in mortality patients across different demographic and geographic groups.

## Results

From 1999 to 2020, the CDC MCD database recorded 59,291 deaths in the US among individuals aged 25 years and older. Among these, deaths in which pneumonia (ICD J18) was listed as the underlying cause of death and DM (ICD E10-E14) was recorded as a multiple cause of death were included in the study (59,291). The crude mortality rate for pneumonia with DM as a contributing cause was 13.3 per 1,000,000 population. Deaths due to causes other than these criteria were excluded.

Among the total deaths analysed, males accounted for 29,021 (48.90%), while females accounted for 30,270 (51.10%). The mortality rate for pneumonia with DM as a contributing cause was higher in females compared to males, indicating a potential demographic disparity. Regarding racial distribution, the highest proportion of deaths occurred among White individuals (n = 48,625, 82%), followed by Black or African American individuals (n = 7,690, 13%), Asian or Pacific Islander individuals (n = 2363, 4%), and American Indian or Alaska Native individuals (n = 613, 1%). The mortality burden was highest among White individuals, highlighting racial disparities in mortality trends related to pneumonia and DM.

A majority of deaths occurred in metropolitan areas (n = 47108, 79.5%), while non-metropolitan areas accounted for (n = 12,183, 20.60%) of deaths. Regarding the place of death, most deaths occurred in medical facilities (n = 42024, 70.90%), followed by decedents' homes (n = 3542, 6.00%), nursing homes or long-term care facilities (n = 11,672, 19.70%), and hospice facilities (n = 1044, 1.8%).

**Figure 1:**
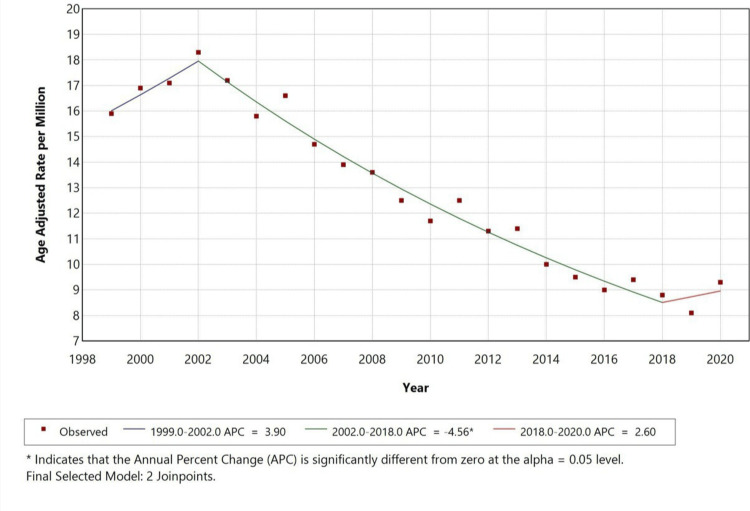
Overall age-adjusted mortality rates among adults aged 25 years and older in the United States, 1999-2020.

From 1999 to 2020, the AAMR for pneumonia with DM as a contributing cause initially showed a rising trend from 1999 to 2002 with an APC of 3.90% (p < 0.05). However, from 2002 to 2018, the AAMR began declining significantly with an APC of -4.56 (p < 0.05). A further rise was observed from 2018 to 2020, with an APC of 2.60% (p < 0.05). This shift suggests a notable change in mortality patterns over the past two decades, with a recent rise in AAMR, as represented in Figure 1.

**Figure 2:**
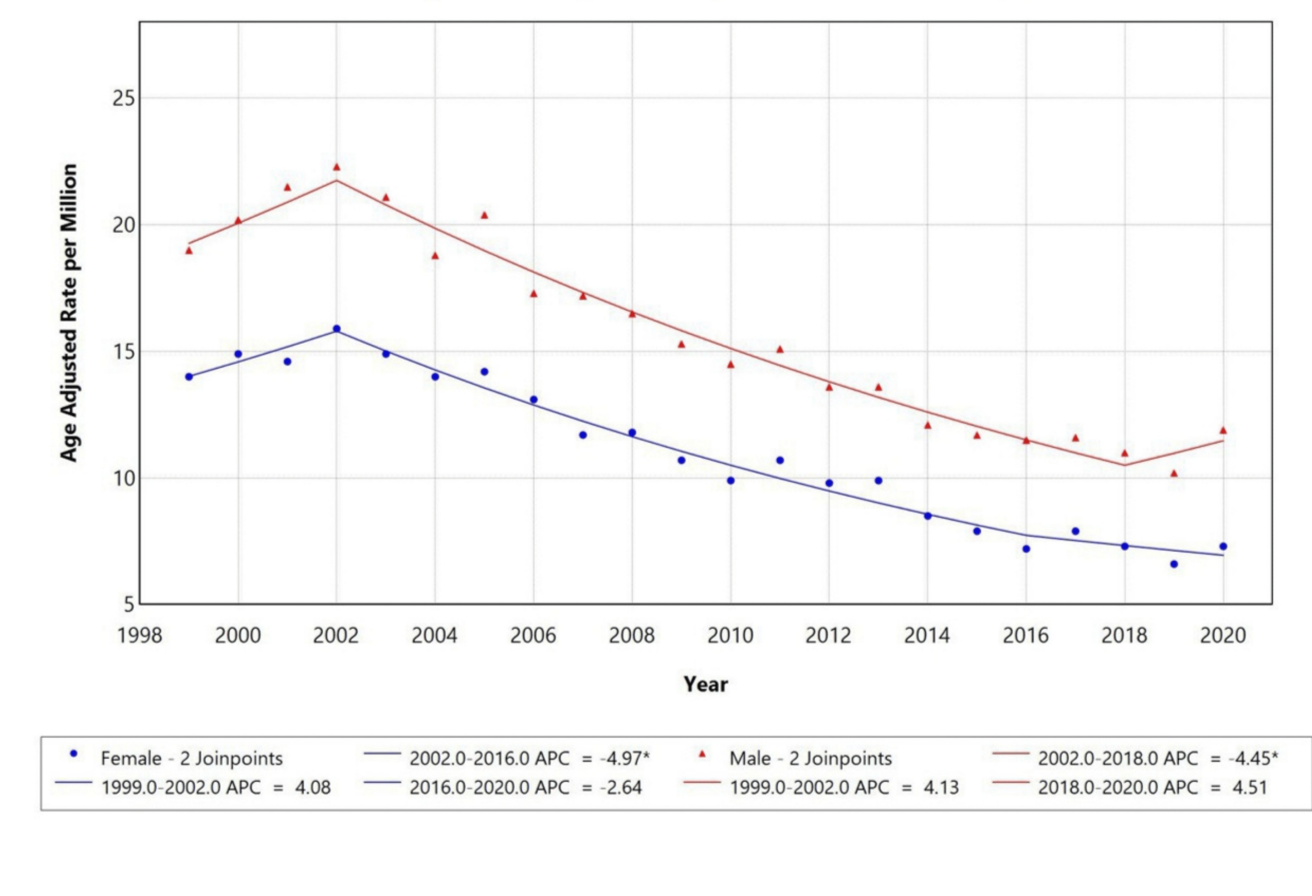
Trends in sex-stratified, age-adjusted mortality rates among adults aged 25 years and older in the United States, 1999-2020. * indicates that the APC is significantly different from zero at the alpha = 0.05 level. APC: annual percentage change

When stratified by gender, females showed a fluctuating trend in AAMR, between 1999 and 2002, the AAMR increased sharply (APC: +4.08%), followed by a significant decline from 2002 to 2016 (APC: -4.97%). After 2013, the AAMR for females began declining again (APC: -2.64%), indicating a resurgence in mortality risk. Temporal trends for males were not displayed due to data suppression for counts <10, limiting reliable trend analysis as represented in Figure 2.

**Figure 3:**
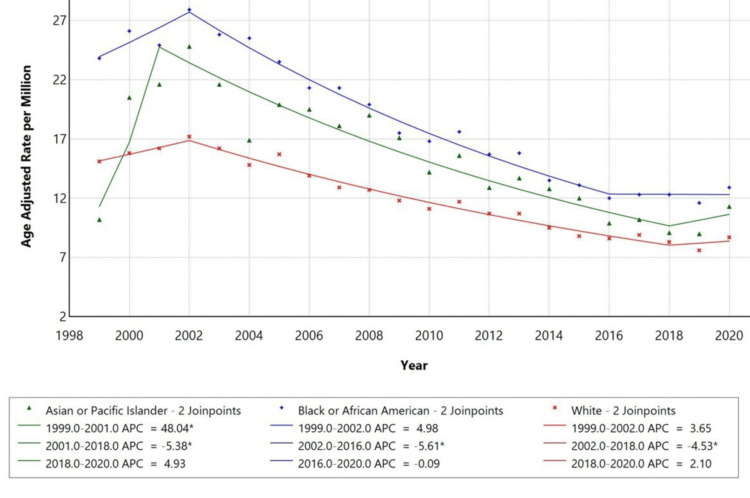
Trends in age-adjusted mortality rates stratified by race among adults aged 25 years and older in the United States, 1999-2020. * indicates that the APC is significantly different from zero at the alpha = 0.05 level. APC: annual percentage change

Racial disparities were observed in pneumonia-related mortality with DM as a contributing cause. Black or African American individuals had the highest AAMR, with a significant increase from 1999 to 2002 (APC: +4.98%). White individuals also demonstrated an increase in AAMR, particularly from 1999 to 2002 (APC: +3.65%), while the later period (2002-2018) showed a decline (APC: -4.53%) as represented in Figure 3. Trends for American Indian/Alaska Native and Asian Pacific Islander populations were not displayed due to data suppression for counts.

## Discussion

This retrospective study analysed mortality trends from 1999 to 2020 using the CDC MCD database, focusing on adults aged 25 years and older in the US, where pneumonia (International Classification of Diseases-10 (ICD-10): J18) was the underlying cause of death and DM (ICD-10: E10-E14) was a contributing cause. A total of 59,291 deaths were reported during this period, with a crude mortality rate of 13.3 per million. Most deaths occurred in metropolitan areas (79.5%), with the majority in medical facilities (70.9%), followed by deaths at homes, hospices, and nursing homes. Mortality was slightly higher in females (51.1%) compared to males (48.9%), and the highest proportion occurred among White individuals (82%), followed by Black and Asian populations. However, despite higher absolute deaths in females and white individuals, AAMR were higher in males and Black populations.

Diabetes can potentially increase the incidence and severity of respiratory infections, including pneumonia, by impairing immune function [10]. Hyperglycaemia alters neutrophil function and reduces cytokine production, increasing susceptibility to infections and worsening outcomes [11]. Moreover, diabetes has been shown to increase the risk of hospitalization, complications, and death from pneumonia [12]. Studies report that individuals with diabetes have up to a threefold higher risk of developing community-acquired pneumonia [13]. They are also more prone to complications like sepsis and respiratory failure [14]. Therefore, it is important to recognize diabetes as a major contributing cause while evaluating mortality trends of pneumonia.

In this study, the overall AAMR for pneumonia with diabetes declined from 16 per million in 1999 to 9 per million in 2020 with a statistically significant APC of -4.56% (p < 0.05). This downward trend indicates meaningful progress in the treatment of diabetes and pneumonia over the past two decades. Similar trends have been observed in a study by Gregg et al., who found a significant decline in infection-related mortality among adults with diabetes, attributed to better glycemic control and preventive care [15]. McDonald et al. also identified reduced mortality in diabetic patients hospitalized with pneumonia, due to earlier diagnosis and treatment [16].

Demographically, males consistently had higher AAMRs than females. In 1999, the AAMR for males was approximately 19 per million, compared to 14 in females. By 2020, these figures dropped to 12 and 7, respectively. APCs were significant for both groups: -4.45 for males and -4.97 for females. The higher mortality in males may be attributed to various biological and behavioural factors. Studies suggest that males have a relatively weaker immune response, with testosterone being linked to immune suppression and estrogen offering some protection [17]. Moreover, men tend to have higher rates of smoking, alcohol usage, and cardiovascular issues, contributing to worse outcomes [18]. Racial disparities were also observed in this study, with Black individuals having the highest AAMR, starting at around 24 per million in 1999 and declining to about 12 by 2020, followed by White people (15 to 8) and Asian or Pacific Islanders experiencing the lowest rates. The decline was significant in both groups, with APCs of -5.61 for Black individuals, -4.53 for White individuals, and -5.38 for Asian or Pacific Islander individuals. The higher mortality among Black individuals is likely due to factors such as higher prevalence of diabetes, poor glycaemic control, and lower vaccination coverage for infections like influenza and pneumococcus [19].

Geographic disparities were also observed, with the majority of deaths occurring in metropolitan areas (79.5%), particularly in large central metros. This is likely due to greater population density, higher baseline disease burden, and more complete reporting. A study found that urban areas may face higher deaths due to poverty concentration, environmental exposures, and fragmented healthcare systems [20]. Furthermore, large urban hospitals may receive more critically ill patients with multiple comorbidities, inflating mortality statistics [21].

Over the 21-year period, AAMR declined significantly across all demographic groups, indicating progress in public health efforts, such as improved vaccination status and more efficient disease management. However, the persistence of demographic and racial disparities despite overall progress suggests that not all populations benefit equally. Further research and targeted strategies are necessary to identify barriers and address inequalities.

Limitations

This study relies on death certificate data, which may be subject to misclassification biases or coding inaccuracies. The MCD database lacks clinical details such as glycaemic control, comorbidities, vaccination status, and treatment history, which could influence pneumonia outcomes. Temporal trends may also be affected by changes in diagnostic or reporting practices over the years. As a retrospective observational study, causality assessment is limited. Additionally, potential confounding variables such as socioeconomic status, healthcare access, and lifestyle factors were not included in the database.

## Conclusions

A total of 59,291 deaths occurred between 1999 and 2020 among adults over 25 years old in the US with both pneumonia and DM. The crude mortality rate was 13.3 per million. Females accounted for a slightly higher proportion (51.1%) than males (48.9%). Racial disparities were evident, with White individuals representing the majority (82%) of deaths, while Black or African American individuals (13%) and other racial groups experienced lower proportions. Geographically, 79.5% of deaths occurred in metropolitan areas, particularly in large central metros (31.1%). Most deaths occurred in medical facilities (66.3%), followed by nursing homes (19.7%). These findings suggest a need for targeted public health strategies in metropolitan regions and among high-risk demographics. Clinicians should prioritize early intervention and integrated care for patients with comorbid pneumonia and diabetes. Future research should explore causal mechanisms behind these disparities and assess the effectiveness of community-based interventions across diverse populations. It should also focus on evaluating the impact of vaccination programs and chronic disease management on mortality trends.

## References

[1] Long ME, Mallampalli RK, Horowitz JC (2022). Pathogenesis of pneumonia and acute lung injury. Clin Sci (Lond).

[2] Sattar SBA, Nguyen AD, Sharma S (2024). Bacterial pneumonia. StatPearls [Internet].

[3] Zhang ZX, Yong Y, Tan WC, Shen L, Ng HS, Fong KY (2018). Prognostic factors for mortality due to pneumonia among adults from different age groups in Singapore and mortality predictions based on PSI and CURB-65. Singapore Med J.

[4] Mizgerd JP (2018). Inflammation and pneumonia: why are some more susceptible than others?. Clin Chest Med.

[5] Banday MZ, Sameer AS, Nissar S (2020). Pathophysiology of diabetes: an overview. Avicenna J Med.

[6] Pan S, Zhang Z, Pang W (2024). The causal relationship between bacterial pneumonia and diabetes: a two-sample mendelian randomization study. Islets.

[7] Olsen MT, Klarskov CK, Hansen KB, Pedersen-Bjergaard U, Kristensen PL (2024). Risk factors at admission of in-hospital dysglycemia, mortality, and readmissions in patients with type 2 diabetes and pneumonia. J Diabetes Complications.

[8] Cilloniz C, Torres A (2024). Diabetes mellitus and pneumococcal pneumonia. Diagnostics (Basel).

[9] (2025). CDC WONDER online database, Multiple Cause of Death files. https://wonder.cdc.gov/controller/datarequest/D77.

[10] Geerlings SE, Hoepelman AI (1999). Immune dysfunction in patients with diabetes mellitus (DM). FEMS Immunol Med Microbiol.

[11] Peleg AY, Weerarathna T, McCarthy JS, Davis TM (2007). Common infections in diabetes: pathogenesis, management and relationship to glycaemic control. Diabetes Metab Res Rev.

[12] Torres A, Peetermans WE, Viegi G, Blasi F (2013). Risk factors for community-acquired pneumonia in adults in Europe: a literature review. Thorax.

[13] Kornum JB, Thomsen RW, Riis A, Lervang HH, Schønheyder HC, Sørensen HT (2008). Diabetes, glycemic control, and risk of hospitalization with pneumonia: a population-based case-control study. Diabetes Care.

[14] Bader MS (2012). Diabetic patients and infections: evaluating the relationship. Postgrad Med.

[15] Gregg EW, Cheng YJ, Srinivasan M, Lin J, Geiss LS, Albright AL, Imperatore G (2018). Trends in cause-specific mortality among adults with and without diagnosed diabetes in the USA: an epidemiological analysis of linked national survey and vital statistics data. Lancet.

[16] McDonald HI, Nitsch D, Millett ER, Sinclair A, Thomas SL (2014). New estimates of the burden of acute community-acquired infections among older people with diabetes mellitus: a retrospective cohort study using linked electronic health records. Diabet Med.

[17] Klein SL, Flanagan KL (2016). Sex differences in immune responses. Nat Rev Immunol.

[18] (2025). Office on smoking and health (OSH). https://www.cdc.gov/tobacco/programs/index.html.

[19] Lu PJ, O'Halloran A, Williams WW, Lindley MC, Farrall S, Bridges CB (2015). Racial and ethnic disparities in vaccination coverage among adult populations in the U.S. Vaccine.

[20] Hale NL, Bennett KJ, Probst JC (2010). Diabetes care and outcomes: disparities across rural America. J Community Health.

[21] Joynt KE, Harris Y, Orav EJ, Jha AK (2011). Quality of care and patient outcomes in critical access rural hospitals. JAMA.

